# "He Was Bought a Bun"

**Published:** 1917-10-13

**Authors:** 


### THE EDITOR'S LETTER-BOX.

### "He was Bought a Bun."

*To the Editor of* THE HOSPITAL.

SIR,—The able writer who signs himself Caduceus, in his article last week on the medical hero, combines a careful style so neatly with freshness of observation and ideas that I hope he will allow me to refer to one of his phrases. It illustrates a point of grammar which in the past has exercised such distinguished writers as Dr. Charles Mercier and Dr. C. Thackray Parsons; but with their letters before me, the principle involved seems still obscure. Caduceus writes, in the course of a good story, "So he was bought, and ate, a bun."

In THE HOSPITAL of June 10, 1916, Dr. Mercier amusingly pilloried the phrase "He was given a pill" as "a horrible perversion" which was "due to the anxiety of writers to use the passive voice," a trick which, of course, care can generally avoid. The following week, in a learned and graceful letter, Dr. Thackray Parsons pointed out that this construction was a common one, and could be paralleled in Shakespeare. (What construction, by the way, could not?) He went on to say that its use was an example of the freedom of the English language, and that the object of a passive verb was known by grammarians as the residual or retained object. He added that it could not be used in any of the inflected languages. A friend showed to me an example of it in Greek the other day. I mention this not to magnify a coincidence, for the exception instances Dr. Parsons' rule, but because I do not trace in his letter any clear principle to justify its use either in inflected languages or in English. Caduceus' sentence "he was bought a bun," following on the Greek example chanced upon the day before, suggests, however, that there may be a principle. Why not state, as a fact, if not as a rule, that in English, as in certain other tongues, a passive verb may, and does, take an object? The simplicity of this does not, I trust, detract from its sufficiency.—Yours faithfully, B. O.

